# Reduction of the blood culture contamination rate in emergency department: a success story

**DOI:** 10.1017/ash.2025.95

**Published:** 2025-09-03

**Authors:** Nor Reah Mustafa, Fida Mohamad, Fauziah Ismail, Maimunah Hasan, Fauzi Idris, Noraida Muhammad, Azrahamiza Abdullah, Sharizan Awang, Nurul Iza Mat Zan, Hakimah Ab Kadir, Wan Noor Faradilla Mat Latif, Che Siti Noraini Abdullah, Yuslizawati Mohd Shaufi, Syaidatull Asmara Aminudden, Silamai A/P Ea Chum, Siti Suraiya

**Affiliations:** 1 Department of Emergency Medicine, School of Medical SciencesUniversiti Sains Malaysia; 2 Infection Control Unit, Hospital USMUniversiti Sains Malaysia; 3 Department of Medical Microbiology and Parasitology, School of Medical SciencesUniversiti Sains Malaysia

**Keywords:** Blood culture contamination, Infection prevention, Emergency department, Quality improvement

## Abstract

**Introduction:** Blood culture result provides a crucial information for patient care. Contaminated blood culture samples may result in inappropriate antimicrobial prescription, increase the cost and unnecessary prolonged hospitalization. In our hospital, the blood culture contamination is high in the emergency department. This initiative aims to improve the emergency department’s blood culture contamination rate which will eventually improve the patient care and benefit the hospital financially. **Methods:** This quality improvement initiative used the Planning, Doing, Checking and Acting (PDCA) models, which provides a simple yet effective approach for problem solving and managing changes. A workgroup consist of Infection control team and emergency department representatives was formed to work on this initiative. Weekly blood culture contamination rate was closely monitored. Root causes were identified, and series of retraining were performed. Blood culture contamination rate before and after the initiative were compared. **Results:** Focus group discussion and site visit reinforcement showed that the high blood culture contamination rate is contributed by many factors. Among the factors included were the inadequacy of blood culture sets, improper use of skin disinfectant, improper hand hygiene techniques and improper aseptic techniques practice by some of the house officers. Blood culture contamination rates 6 months before and during feedback intervention showed significant decrease (3.52% before intervention and 2.95% after intervention; *P* < .05. **Discussion:** Blood culture contamination rate reduced significantly after the joint initiative continued to decrease with the use of a predisinfection process with 2% Chlorhexidine gluconate cloth before blood sample collection process. Practice improvement also was evident with effective feedback mechanism.

Conclusion:

